# Early diagnosis with sequencing and successful treatment of *Bipolaris* prosthetic valve endocarditis

**DOI:** 10.1016/j.mmcr.2022.02.001

**Published:** 2022-02-13

**Authors:** Emily A. Shephard, Julia Sapozhnikov, Ziv Beckerman, Donald K. Murphey

**Affiliations:** a Dell Seton Medical Center, 1500 Red River St, Austin, 7870, United States; b Dell Children's Medical Center, 4900 Mueller Blvd, Austin, 78723, United States; c Dell Medical School, 1501 Red River St, Austin, 78712, United States

**Keywords:** Fungal endocarditis, *Bipolaris*, Dematiaceous mold, Endocarditis

## Abstract

We present a case of a 48-year-old man with congenital bicuspid aortic valve, history of Ross procedure, prosthetic pulmonary valve and homograft with rapid molecular diagnosis and prompt surgical and medical treatment for *Bipolaris* fungal endocarditis with excellent outcome with early valve replacement, debridement, combination antifungal therapy, ongoing suppressive therapy after treatment.

## 1 Introduction

*Bipolaris* and *Curvularia* are closely related, dark-walled, dematiaceous mold species usually found in the environment. These organisms have been reported to cause a variety of infections, including skin and soft tissue infections, brain abscesses, and sinusitis. Infections caused by this group of molds are termed *Phaeohyphomycosis*. The general approach to treatment of these infections includes Amphotericin B for severe infections and prolonged durations of azole antifungals, however, optimal therapy for these infections is not well understood [1]. We present a case of prosthetic valve endocarditis caused by *Bipolaris* species, with a rapid diagnosis and aggressive treatment resulting in clinical improvement.

## 2 Case presentation

A 48-year-old male with a history of congenital bicuspid aortic valve and severe aortic stenosis presented with shortness of breath, activity intolerance and back pain. Surgical history was significant for a Ross procedure and repair of aortic aneurysm with Dacron graft in 2002, followed by pulmonary homograft replacement in 2018 due to homograft stenosis. Subsequently, he underwent repeat replacement of his right-ventricle to pulmonary artery (RV-PA) conduit with a porcine valved conduit due to early homograft degeneration 4 ½ months before the current presentation. Approximately 3 months prior to presentation (1.5 months after his last operation), the patient was admitted with fever and chest pain. Endocarditis workup was unrevealing. CTA demonstrated a right lower lobe posterior segmental pulmonary embolism; the patient was discharged with anticoagulation treatment.

Three weeks later, the patient was re-admitted with dyspnea. On the day of admission (day 0) he had stable hemodynamics, laboratory findings were significant for WBC count of 8100 cells/mL with 77.2% neutrophils, C-reactive protein of 2.3 mg/dL and erythrocyte sedimentation rate (ESR) of 71 mm/hr. Echocardiogram demonstrated severe RV-PA conduit stenosis with high suspicion for endocarditis, dilated right ventricle with mildly decreased function. Abdominal ultrasound showed splenomegaly, repeat CT angiography of the chest had stable old embolus, with no new changes, CNS MRI had a tiny focus of microhemorrhage in the left thalamus, and CNS CT angiography was unremarkable. An eye exam was normal. His social history was significant for pet cat exposure, gardening, and history of very remote travel to Mexico with spelunking in bat caves. The patient resides in central Texas. He has no history of substance abuse or immune deficiency. Empiric antibiotic therapy was initiated with vancomycin and cefepime.

Infectious workup included blood cultures, acid-fast bacilli (AFB) and fungal blood cultures, 1,3-Beta D Glucan, histoplasma and coccidiomycosis antibodies, T-spot, *Bartonella* titers, *Brucella* titers, Q-fever titers, and a next-generation sequencing on blood - Karius ® (Redwood City, CA).

On the third day of admission, the patient's Karius resulted with *Curvularia papendorfii (Bipolaris)* and *Curvularia lunata* at 364 DNA molecules per microliter (MPM) and at 101 MPM, respectively (reference range for both <10 MPM). Blood cultures remained negative. 1,3-Beta D Glucan was elevated at > 500 pg/mL. Vancomycin and cefepime were discontinued, and Amphotericin B liposomal 5 mg/kg daily and voriconazole 6mg/kg IV every 12 hours followed by 4 mg/kg IV every 12 hours were initiated. The patient also underwent transesophageal echocardiogram which showed a dilated right ventricle with impaired systolic function, a dilated right atrium, moderate tricuspid regurgitation, narrowing of the bioprosthetic pulmonary valve with stenosis, and trace aortic regurgitation. On the same day, the patient experienced a vasovagal event and required chest compressions. Due to concerns about fungal endocarditis involving a prosthetic valve, he was taken urgently to the operating room. During surgery, the graft was noted to be severely infected, and it was replaced with a new pulmonary homograft.

**Fig. 1:**
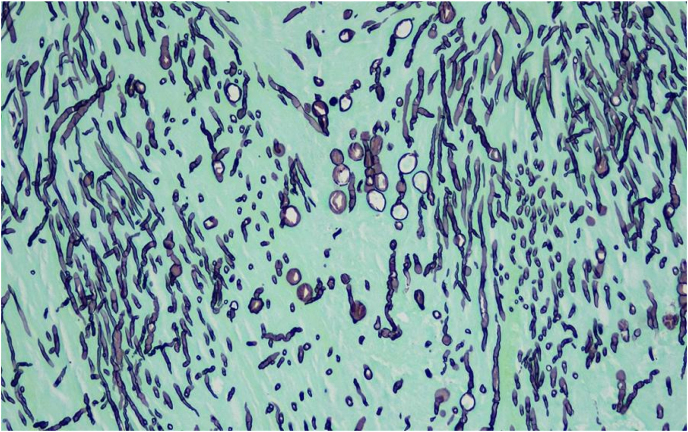
Bipolaris in cardiac tissue shown with Grocott's Methenamine Silver Stain (GMS).

After two days of incubation, fungus was isolated from both the infected graft material and surgical tissue cultures and identified as *Bipolaris* species (see Fig. 1). Fungal susceptibilities were obtained using broth microdilution (see Table 1) from ARUP reference laboratory. Other evaluations for infectious endocarditis remained negative.

**Table 1:** *Bipolaris* species Antifungal Susceptibilities.

Antifungal Tested	MIC (microgram/mL)
Amphotericin B	1
Anidulafungin	4
Caspofungin	≥8
Isavuconazole	2
Itraconazole	0.5
Micafungin	≥8
Posaconazole	0.25
Voriconazole	0.5

His hospital course was complicated by *Clostridiodes difficile* infection identified on day 12 of hospitalization. He was treated with 10 days of oral vancomycin and improved.

Routine and fungal blood cultures were negative. Repeat Karius testing was obtained on day 28 of hospitalization and identified no organisms. Repeat 1,3-Beta D Glucan on day 28 was improving but remained elevated at 111 pg/mL. C-reactive protein increased early to 7.0 mg/dL, then decreased to 1.5 mg/dL later in his therapy.

Based on fungal susceptibilities and therapeutic drug monitoring with target levels of >1 mg/L posaconazole and 1–5.5 mg/L for voriconazole, the patient's voriconazole was switched to posaconazole 300 mg every 12 hours for 2 doses then 300mg daily; however, the patient developed persistent significant anorexia, nausea and vomiting. The patient was switched back to voriconazole therapy and those symptoms improved.

Amphotericin dosing was reduced to 3mg/kg due to nephrotoxicity with elevated serum creatinine to 2.1 mg/dL. Pulmonary edema and pleural effusions limited our ability to give large amounts of fluids. Amphotericin was decreased from daily to thrice weekly, then increased back to daily based on tolerability. This was complicated by rechallenge with posaconazole resulting in nausea and vomiting again. Voriconazole was switched to isavuconazole (372mg every 8 hours for 6 doses, then 372mg once daily) at week five of therapy due to QTc prolongation.

The patient completed 6 weeks of Amphotericin B liposomal with concurrent voriconazole, posaconazole and isavuconazole and was discharged on lifelong isavuconazole suppression therapy.

## 3 Discussion

*Curvularia* and *Bipolaris* are uncommon pathogens, with less than ten cases of endocarditis with these pathogens reported in the literature. Here, we present a case of *Bipolaris* endocarditis. Our case highlights the utility of advanced diagnostic techniques that allowed us to make a diagnosis quickly, and a successful treatment outcome utilizing intensive induction therapy and aggressive surgical intervention. The rapid identification by next generation sequencing found *Curvularia* while valve cultures grew *Bipolaris.* This difference is likely due to limitations in molecular identification. We assume our national microbiology reference lab identification is correct. We were not able to sequence this isolate. Interestingly, our patient had pre-existing pulmonary calcified granulomas from old histoplasmosis that was unrelated to his current illness.

There are few concrete treatment recommendations for fungal endocarditis, and mortality is often high [2]. Infective endocarditis guidelines recommend that surgical intervention and empiric treatment with amphotericin B should be mainstays of treatment for fungal endocarditis. Additionally, guidelines recommend induction treatment with amphotericin B, then maintenance therapy with (often lifelong) azole therapy [2]. While these recommendations do not specifically address therapy for endocarditis with dematiaceous molds, it seems reasonable to employ this treatment strategy for any fungal endocarditis. We chose to continue liposomal amphotericin B and voriconazole for induction therapy, both agents for 6 weeks, followed by isavuconazole indefinitely as secondary prophylaxis. Our patient experienced nausea and vomiting with posaconazole on two attempts.

*Bipolaris and Curvularia* have been reported in few cases of endocarditis previously in the literature. The first report of *Curvularia* endocarditis was published in 1970, where *Curvularia* was identified as the causative agent in a case of prosthetic valve endocarditis that was diagnosed post-mortem. The patient in this case was treated empirically with antibiotics but received no antifungals, and surgical intervention was delayed by almost two weeks after the patient's admission [3]. In another fatal case of endocarditis, the causative organism was not identified as *Bipolaris* until post-mortem examination of a vegetation, and the patient only received empiric antibiotics. The patient had fungal growth on 2 of 23 blood cultures, but antifungals were not started due to unclear etiology of the patient's infection [4]. Both of these cases demonstrated difficulties with diagnosis of this organism.

Other descriptions of endocarditis with *Curvularia* have described successful treatment with prompt surgical intervention and amphotericin B in combination with voriconazole, ketoconazole, and terbinafine [[5], [6], [7], [8]]. Our case highlights the importance of rapid molecular identification of the causative pathogen, aggressive medical and surgical therapy, and a successful outcome using combination antifungal therapy for an uncommon pathogen.

## Ethical Form

Please note that this journal requires full disclosure of all sources of funding and potential conflicts of interest. The journal also requires a declaration that the author(s) have obtained written and signed consent to publish the case report/case series from the patient(s) or legal guardian(s).

The statements on funding, conflict of interest and consent need to be submitted via our Ethical Form that can be downloaded from the submission site www.ees.elsevier.com/mmcr. **Please note that your manuscript will not be considered for publication until the signed Ethical Form has been received.**

## Declaration of competing interest

There are none.
